# Structure-based machine-guided mapping of amyloid sequence space reveals uncharted sequence clusters with higher solubilities

**DOI:** 10.1038/s41467-020-17207-3

**Published:** 2020-07-03

**Authors:** Nikolaos Louros, Gabriele Orlando, Matthias De Vleeschouwer, Frederic Rousseau, Joost Schymkowitz

**Affiliations:** 1 Switch Laboratory, VIB Center for Brain and Disease Research, Herestraat 49, 3000 Leuven, Belgium; 2 Switch Laboratory, Department of Cellular and Molecular Medicine, KU Leuven, Herestraat 49, 3000 Leuven, Belgium

**Keywords:** Molecular modelling, Molecular biophysics, Computational biology and bioinformatics, Machine learning, Molecular biology

## Abstract

The amyloid conformation can be adopted by a variety of sequences, but the precise boundaries of amyloid sequence space are still unclear. The currently charted amyloid sequence space is strongly biased towards hydrophobic, beta-sheet prone sequences that form the core of globular proteins and by Q/N/Y rich yeast prions. Here, we took advantage of the increasing amount of high-resolution structural information on amyloid cores currently available in the protein databank to implement a machine learning approach, named Cordax (https://cordax.switchlab.org), that explores amyloid sequence beyond its current boundaries. Clustering by t-Distributed Stochastic Neighbour Embedding (t-SNE) shows how our approach resulted in an expansion away from hydrophobic amyloid sequences towards clusters of lower aliphatic content and higher charge, or regions of helical and disordered propensities. These clusters uncouple amyloid propensity from solubility representing sequence flavours compatible with surface-exposed patches in globular proteins, functional amyloids or sequences associated to liquid-liquid phase transitions.

## Abstract (web summary)

An increasing number of amyloid structures are determined. Here, the authors present the structure-based amyloid core sequence prediction method Cordax that is based on machine learning and allows the detection of aggregation-prone regions with higher solubility, disorder and surface exposure, and furthermore predicts the structural topology, orientation and overall architecture of the resulting putative fibril core.

## Introduction

The amyloid cross-*β* state is a polypeptide conformation that is adopted by 36 proteins or peptides associated to human protein deposition pathologies^1^. It also constitutes the structural core of a growing number of functional amyloids in both bacteria and eukaryotes^2,3^. Beyond these bona fide functional and pathological amyloids it has been demonstrated that many if not most proteins can adopt an amyloid-like conformation upon unfolding/misfolding^4^. This has led to the notion that just like the α-helix or *β*-sheet, the amyloid state is a generic polypeptide backbone conformation but also that amino acids have different propensities to adopt the amyloid conformation^5^. Initially, it was observed that amyloid-like aggregation correlates with hydrophobicity, *β*-strand propensity, and (lack of) net charge^6^. This triggered the development of aggregation prediction algorithms that essentially evaluate the above biophysical propensities^7,8^. Others extended to scaling residue propensities between protein folding and aggregation^9,10^. These algorithms confirmed the ubiquity of amyloid-like propensity in natural protein sequences and particularly in globular proteins as it was estimated that 15–20% of residues in a typical globular domain are within aggregation-prone regions (APRs)^11,12^. These APRs are sequence segments of six to seven amino acids in length on average and are mostly buried within the protein structure where they constitute the hydrophobic core stabilising tertiary protein structure^13–15^. On the other hand, the increasing identification of both yeast prions and functional amyloids clearly indicated that amyloid sequence space is not monolithic and that more polar/less aliphatic sequences represent important alternative populations of amyloid sequence space^3^. The limited sensitivity of the above cited algorithms to specifically identify these other subpopulations confirmed the underestimated sequence versatility of the amyloid conformation. Indeed, more recently the role of amyloid-like sequences in proteins mediating liquid–liquid phase transitions again demonstrates the ubiquity of the amyloid in biological function and further withers the image of the amyloid state as a predominantly disease and/or toxicity-associated protein conformation^16–18^. Rather, this suggests that like globular protein folding, amyloid assembly is a matter of kinetic and thermodynamic control that can be evolutionary tuned by sequence variation and selection.

Efforts to develop aggregation predictors that can identify a broader spectrum of amyloid sequences have increased over the years^19^. Such approaches focused on identifying position-specific patterns by reference to accumulated experimental data of APRs^20–22^, or by using energy functions of cross-beta pairings^23^. Recently developed meta-predictors produce consensus outputs by combining previous methods, in an attempt to boost performance^24,25^. Indirect structure-based methods were initially developed by considering secondary structure propensities^26,27^. Complementary studies extended this notion by suggesting that disease-related amyloids form β-strand-loop-β-strand motifs^28^. However, the principle of using structural information to accurately predict aggregation prone segments in protein sequences stems from the detailed work of Eisenberg and co-workers. The 3D-profiling method utilised the crystal structure of the fibril-forming segment NNQQNY (PDB ID: 1YJO) derived from the Sup35 prion protein, to thread and evaluate sequence fitting using the Rosetta energy function^29^. In this work, we build on this principle to develop Cordax, an exhaustively trained regression model that leverages a substantial library of curated template structures combined with machine learning. Cordax not only detects APRs in proteins, but also predicts the structural topology, orientation and overall architecture of the resulting putative fibril core. To validate the accuracy of our predictions, we designed a screen of 96 newly predicted APRs and experimentally determined their aggregation properties. Using this approach, we identified less hydrophobic polar and charged aggregation prone sequences that increasingly uncouple solubility and amyloid propensity, closely resembling characteristics of phase-separation inducers. Clustering by t-distributed stochastic neighbour embedding reveals the heterogeneous substructure of amyloid sequence space consisting in varying clusters corresponding to sequences compatible with globular structure, functional scaffolding amyloids, N/Q/Y-rich prions, helical peptides and intrinsically disordered sequences. Together, the structural exploration performed here demonstrates that the field now gathered sufficient structural and sequence information to start classifying amyloids according to different structural and functional niches. Just like for globular proteins in the 1980s, this will allow to fine-tune both general and context-dependent structural rule learning allowing to manipulate and design amyloid structure and function.

## Results

### Overall approach of Cordax

We wanted to design a novel structure-based amyloid core sequence prediction method that (a) leverages all the available structure information that is currently available, and (b) employs a machine-learning element for optimal prediction performance. To this end, we first built a curated template library of amyloid core structures as described in the paragraph below. In the vein of previous prediction methods^29^, we fixed on the hexapeptide as a unit of prediction. In order to determine the amyloid propensity of a query hexapeptide we start by modelling its side chains on all the available amyloid template structures using the FoldX force field^30^, which yields a model and an associated free energy estimate (Δ*G*, kcal/mol) for each template. These free energies are then fed into a logistic regression model, which is a simple statistical method relating a binary outcome to continuous variables. The prediction output of Cordax is multiple: First, there is the prediction from the logistic regression whether or not the segment is an amyloid core sequence. Second, for the sequences deemed amyloid core, the most likely amyloid core model is provided. For longer query sequences, a sliding window approach is adopted. The technical details of the pipeline can be found in the “Methods” section.

### Refinement of fibril structures for machine learning

**Fig. 1:**
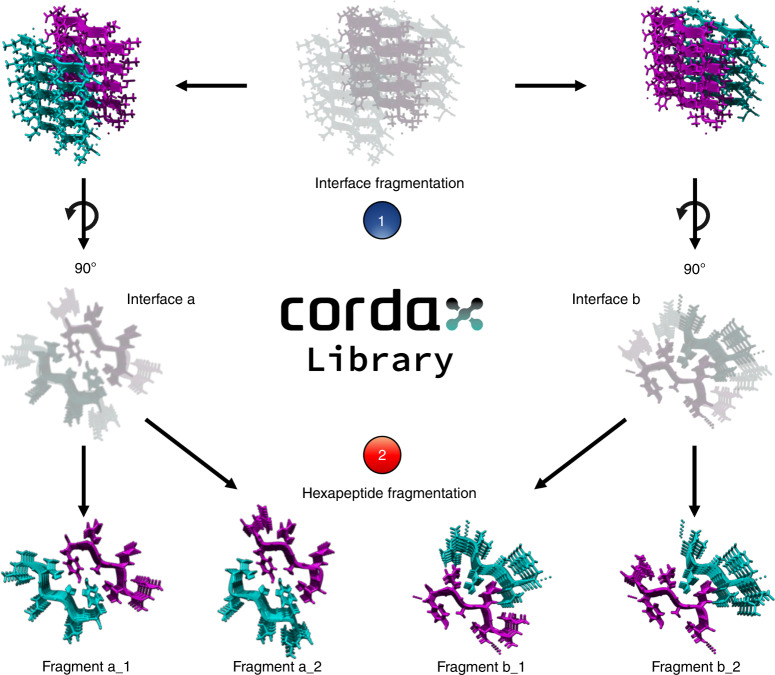
Processing steps of the peptide fragment library. **a** Crystal contact information was used to generate fibril cores from isolated PDB structures. Structures containing multiple packing interfaces were split into individual templates (1), which were in turn split into hexapeptide core fragments (2). Source data are provided as a Source Data file.

We isolated 78 short segment fibril core high-resolution structures from the Protein Data Bank (Supplementary Data 1). Templates were grouped into seven distinct topological classes out of eight theoretically possible based on their overall structural properties, as previously proposed by Sawaya et al.^31^. Briefly, topologies are defined by whether *β*-sheets have parallel versus antiparallel orientation, by the orientation of the strand faces that form the steric zipper (face-to-face versus face-to-back), and finally the orientation of both sheets towards each other and whether that results in identical or different fibril edges. This complexity was addressed by generating an ensemble of amyloid cores per structure using crystal contact information derived from the solved structures. Every template comprises two facing *β*-sheets, each composed of five successive *β*-strands. Since parallel architectures can share more than one homotypic packing interface, those structures were split into separate individual entries (Fig. 1). To ensure uniformity, we expanded the number of structural variants by breaking down longer segments into hexapeptide constituents, thus yielding a library of 179 peptide fragment structures (Fig. 1 and Supplementary Data 1).

**Fig. 2:**
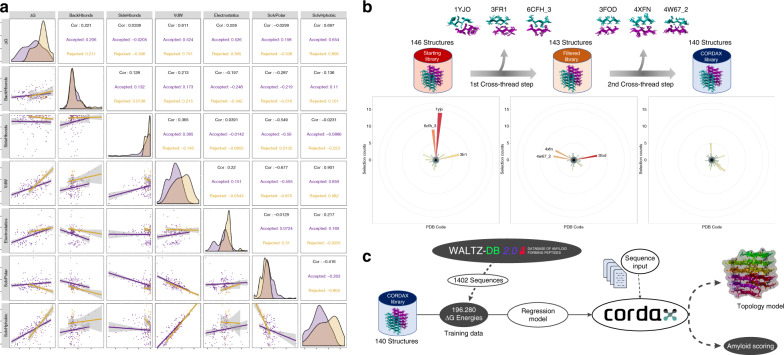
Optimising the Cordax structural library. **a** Correlation plot of interface energies calculated using FoldX. Top half shows correlation values with scatter plots indicated at the bottom half. Rejected fragments sharing low shape complementarity (shown in yellow) have correlating weak van der Waals interfaces, as well as poor solvation energies for hydrophobic side chains compared to the remaining library (indicated in purple). Linear regression lines are shown with 95% confidence interval (shown in grey shaded areas). **b** Promiscuity sorting of the structural library performed as a two-step cross-threading process. Circular histograms highlight three major promiscuous structures (*n* > 5) which were removed during the primary (PDB ID: 1YJO, 3FR1 and 6CFH_3) and secondary step (PDB ID: 3FOD, 4XFN and 4W67_2). **c** Schematic representation of Cordax training and the derived pipeline.

The amyloid interaction interfaces were analysed in detail following energy refinement by the FoldX force field^30^. During this step we identified and rejected 33 imperfect *β*-packing interfaces formed by *β*-strands that contribute less than three interacting residues, thus reducing the ensemble to 146 structures (Supplementary Data 1). Detailed analysis of the contributions of various energy components showed that these excluded *β*-packing interfaces have inefficient shape complementarity and low overall stability, stemming from a combination of weak electrostatic contributions, diminished van der Waals interactions and exposure of hydrophobic residues to the solvent (Fig. 2a).

Previous work has highlighted that distinct topological layouts can potentially introduce a stronger tolerance for the integration of protein sequence segments and as a result can generate several potential type-I errors (false positives)^29^. To address this issue, we implemented a two-step cross-threading exploration of putative structural promiscuous traps. In more detail, we extracted a non-redundant set of hexapeptide sequences from the structural library (73 sequences), which was subsequently cross-modelled in an all-against-all reiteration process. Using an empirical cut-off threshold (=5), a sum of three structural fragments was initially identified and removed. Eliminating these structures led to the identification and subsequent elimination of three additional promiscuous templates, resulting in the final Cordax library, composed of 140 zipper structures (Fig. 2b and c).

### Benchmarking aggregation propensity detection with Cordax

**Fig. 3:**
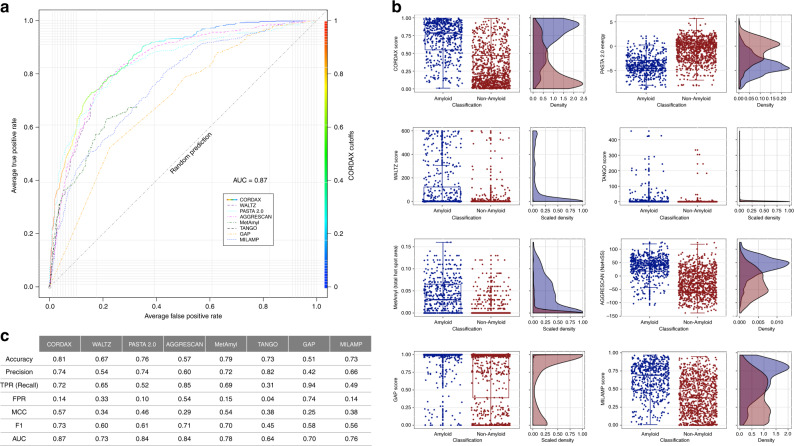
Benchmarking of CORDAX. **a** ROC curve analysis for Cordax and seven other state-of-art methods against WALTZ-DB 2.0. For WALTZ, TANGO and MetAmyl, FPR stops at earlier rates due to minimal scoring variations. **b** Cordax score distribution compared to other tools. The regression model achieves better scoring separation for predictions between amyloid-forming (shown in blue) and non-amyloid sequences (shown in red). Distributions are shown as standard box-plots highlighting median, upper (Q3) and lower (Q1) quartiles. Whiskers indicate the ranges for values within 1.5 interquartile range (IQR) above and below Q3 and Q1, respectively. Density plots for WALTZ, TANGO, MetAmyl and GAP are scaled due to the overrepresentation of unscored values or false positives, respectively. **c** Performance metrics comparison indicating Cordax superiority to other sequence predictors (MCC = 0.57, F1 = 0.73 and AUC = 0.87).

As an initial test of the prediction accuracy of the regression model, we performed leave-one-out cross-validation on the training dataset^32^ and performance metrics were determined on a peptide basis. Due to the extensive size of the dataset, comparison to other software was performed only with methods supporting multiple sequence input and a non-binary scoring function, since performances were compared using receiver operating characteristic (ROC) analysis^33^. The ROC curves generated highlight that Cordax performance exceeds over seven state-of-the-art methods, which we applied using optimised options defined by the developers^7,9,21–24,34^. In detail, Cordax performs well over random as depicted by the highest total area under the curve (AUC) value of 0.87 (Fig. 3a). Distribution analysis of the scoring values indicates that the method achieves optimal separation, resulting in minimal scoring overlay between positive and negative amyloid forming sequences (Fig. 3b). As previously reported, TANGO showed high specificity due to the overrepresentation of unscored values, which is also evident for WALTZ as well as MetAmyl, which incorporates the latter method in its meta-prediction. The cost of high specificity is also reflected by the calculated F1 values, as PASTA and TANGO report low recall values. On the other hand, AGGRESCAN and GAP produce significant overpredictions as depicted by their reported false-positive rates (FPR values of 0.54 and 0.76, respectively) (Fig. 3c). The optimal score thresholding of our method was determined from the ROC curve analysis as the score where predictions show the highest sensitivity-to-specificity ratio. According to this, Cordax achieves a well-balanced prediction by reporting with high specificity (86%) more than 7 out of 10 aggregation prone segments (72%), which is reflected by the highest calculated MCC, AUC and F1 values compared to other available software (Fig. 3c).

To further benchmark the method, we tested it against full-length protein sequences. For this we used a standardised set of 34 annotated amyloidogenic proteins that was previously implemented for validation of several previous aggregation predictors^25^, following a filtering step for potential overlaps to the training data set. Despite its wide use, this collection suffers from insufficient experimental characterisation of certain large entries (i.e. gelsolin, kerato-epithelin, lactoferrin, amphoterin and others), which has been shown to introduce type-I errors (false positives). This error propensity derives from non-amyloid annotations which primarily correspond to regions of undetermined aggregation propensity, a notion that is highlighted by recent studies, such as in the case of calcitonin^35^, cystatin-C^36^ and transthyretin^37^. In contrast, other proteins have been linked to the formation of *β*-helical structures and as an after effect contain elongated fragments characterised, yet unverified in their entirety, as amyloidogenic, which can introduce type-II errors (false negatives) when applying predictors of local aggregation propensity^38–41^. The aforementioned shortcomings are reflected by the low MCC values that are reported for all aggregation predictors (Supplementary Table 1) and the fact that predicted segments were originally considered neutral, but later shown to be aggregation hotspots (Supplementary Fig. 1)^35–41^.

### Designed APR nucleators validate the accuracy of Cordax predictions

**Fig. 4:**
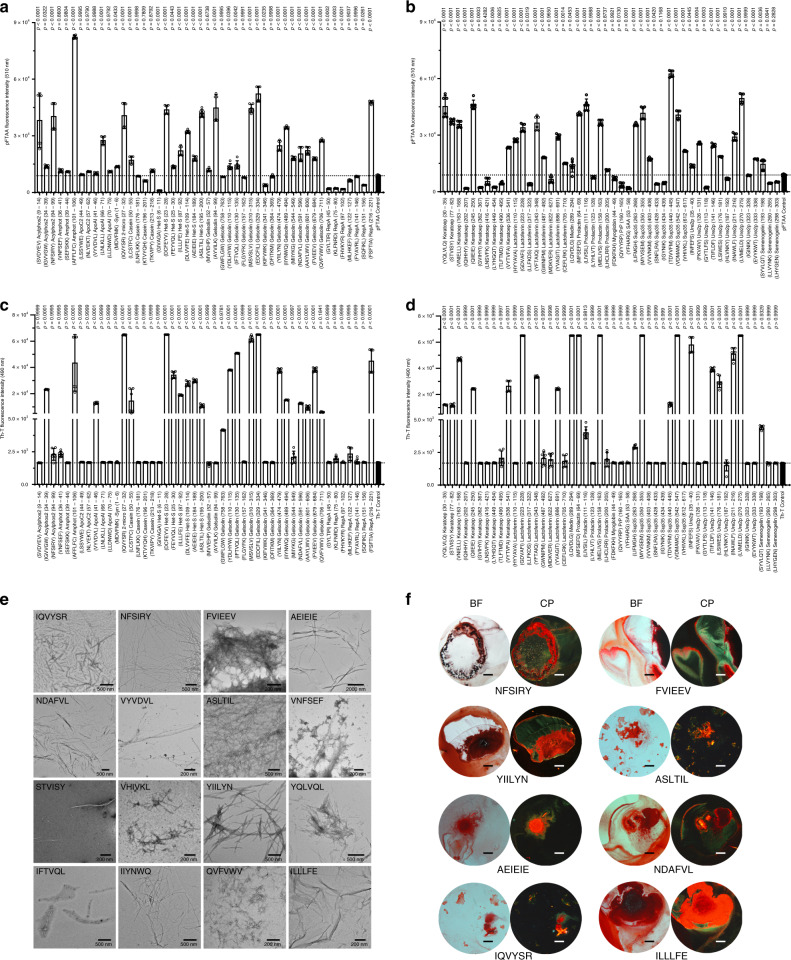
Amyloid-forming properties of the peptide screen designed by employing Cordax. **a** and **b** Measured pFTAA and **c** and **d** Th-T fluorescence of synthetic peptides following rotation at 200 μM for 5 days. Data represents mean ± SD (*n* = 6 independent experiments, statistics: one-way ANOVA with multiple comparison against the vehicle control). **e** Electron micrographs of amyloid fibrils formed by Th-T or pFTAA-binding peptides. **f** Suspensions of amyloid fibrils bind Congo red as displayed under bright field illumination (BF) and exhibit typical for amyloids apple-green birefringence under crossed-polarised light (CP). Scale bars: 500 μm. Source data are provided as a Source Data file.

In the interest of improving the current description of the familiar amyloidogenic protein dataset, we selected and synthesised a subset of 96 peptides corresponding to strong aggregation prone regions identified in these proteins by Cordax. Apart of prediction strength, the peptide screen was also selectively constructed to ensure broad sequence variability and a wide distribution on the proteins of the dataset, with a preference for longer entries defined by inadequate previous characterisation. Peptide sequences were cross-checked and filtered to exclude overlapping sequences with previously identified amyloid regions and WALTZ-DB (Supplementary Data 2). The remaining selection of 96 peptides were synthesised using standard solid phase synthesis and their amyloid-forming properties were initially examined using Thioflavin-T (Th-T) or pFTAA binding, following rotating incubation for 5 days at room temperature. The binding assays are complementary, as Th-T and pFTAA are opposingly charged molecules, which increases the amyloid identification rate by overcoming cases of dye-specific failure to bind to amyloid surfaces based on charge repulsion. Under these conditions, 66 peptides successfully bind to the specific dyes (Fig. 4a and b) by forming fibrils with typical amyloid morphologies and properties that were verified using transmission electron microscopy (Fig. 4c) and Congo red staining for selected cases (Fig. 4d). As these dyes are known to yield false negatives, in particular for short peptides, all dye-negative peptides were further investigated using electron microscopy. During this scan, we recovered 19 additional sequences that were capable of forming sparse amyloid-like fibrils with shorter lengths (Supplementary Fig. 2). Taking the latter into account, Cordax was able to fish out a total number of 85 novel nucleation segments with unparalleled accuracy (89%), thus providing a rigorously improved description of the protein set to be used for the efficient testing and development of future predictors (Supplementary Fig. 1).

### Cordax detects highly soluble surface-exposed conformational switches

**Fig. 5:**
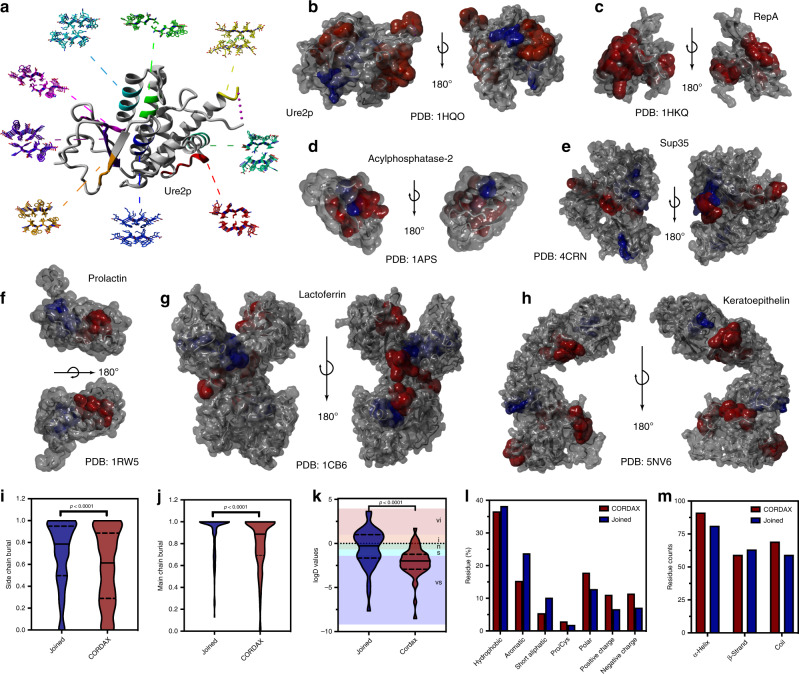
Cordax identifies surface-exposed aggregation nucleators spanning residues that are typically considered unconventional for amyloid fibril formation. **a** Schematic representation of Cordax-predicted topological models for APRs charted against the cognate native crystal structure of the amyloidogenic protein Ure2p. **b**–**h** Surface representation of folded structures for **b** Ure2p, **c** RepA, **d** Acylphosphatase-2, **e** Sup35, **f** Prolactin, **g** Lactoferrin and **h** Kerato-epithelin reveals that aggregation nucleators uniquely identified by Cordax (highlighted in red) are primarily exposed to the surface of proteins, compared to segments of joint prediction (shown in blue) which are predominantly buried within the hydrophobic core of the native fold. Cordax-specific predicted APRs produced lower volumetric burial values, calculated using FoldX, for **i** side chain and **j** main chain groups indicating that they are considerably exposed compared to jointly identified nucleators. **k** Partition coefficients indicate that Cordax-specific APRs are significantly more soluble compared to typically predicted sequences that are primarily hydrophobic and therefore insoluble. Solubility regions (vi very insoluble, i insoluble, n neutral, s soluble, vs very soluble) are shown as coloured backgrounds^72^. Significant differences were computed using one-way ANOVA with multiple comparison. **l** Surface-exposed Cordax-specific APRs are composed of residues with a 20% increase in polar and charged side chains, in expense of hydrophobic residues (*n* = 219 residues in APRs identified by Cordax, *n* = 203 residues in APRs from joined predictions). **m** Secondary structure analysis, using FoldX, indicates that Cordax identifies several APRs that reside in α-helical or unstructured regions within the native fold, suggesting that amyloidogenic proteins may harbour a plethora of exposed conformation switches that can act as potential nucleators of amyloid fibril formation, under suitable misfolding conditions (*n* = 219 residues in APRs identified by Cordax, *n* = 203 residues in APRs from joined predictions). Violin plots represent the kernel probability densities of the data with the median, upper and bottom quartiles. Source data are provided as a Source Data file.

The expanded amyloidogenic annotation of the protein dataset was supplemented with structural analysis of the newly identified aggregation prone regions. Out of 96 peptides designed and experimentally tested, 85 peptides were found to display evident amyloid-forming features, with more than half (55.3%) being predicted specifically by Cordax, contrary to shared predictions with sequence-based tools of high specificity (44.7%) (Supplementary Data 2). Pinpointing the location of the identified nucleators in parental protein folds (Fig. 5a) revealed that APRs picked up both by Cordax and traditional sequence-based methods are usually found buried within the core of soluble proteins. Contrary to what has been previously reported^14,15^, however, our regression model also discovered additional nucleating sequences that primarily appear to reside on the surface of protein molecules (Fig. 5b–h) and as a result, are characterised by high solvent exposure (Fig. 5i and j). Partition coefficients clearly indicate that these exposed peptide segments identified by Cordax are primarily water-soluble sequences, whereas APRs that are predicted by the majority of sequence-based predictors are largely insoluble (Fig. 5k). Sequence distribution analysis signifies that this increased exposure and solubility is complemented by an expected decrease in sequence hydrophobicity (Fig. 5l). More specifically, APRs identified solely by Cordax are relatively enriched in charged or polar side chains (Fig. 5l) and are frequently parts of α-helical or unstructured segments (Fig. 5m). This implies that these regions are in fact conformational switches that may, under fitting misfolding conditions, transiently move towards the formation of *β*-aggregates. The fact that these sequences are not dictated by typical sequence propensities, such as hydrophobicity or *β*-structure tendency, explains why sequence-based predictors overlook them.

### Cordax infiltrates uncharted areas of amyloid sequence space

**Fig. 6:**
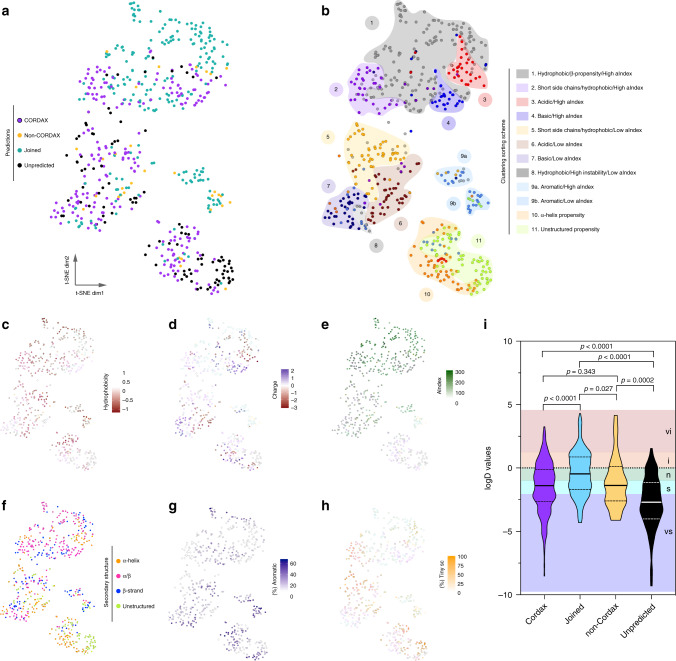
t-SNE 2D-representation of the known experimentally determined amyloidogenic sequence space. **a** State-of-the-art sequence-based methods predict amyloid sequences, with (shown in cyan) or without Cordax (shown in yellow), that are grouped together in a major landing cluster and two islands. Cordax predictions (shown in purple) transgress towards areas of amyloid-forming sequences that remain undetected by most methods (shown in black). **b** Clustering of the t-SNE map using basic physicochemical properties and amino acid composition of the amyloid peptides. Each data point is colour-coded based on the sorting scheme shown in the legend and background areas are used to pinpoint the major clusters of each defined category. The clustering scheme was defined by characterising the t-SNE map using peptide **c** hydrophobicity, **d** net charge, **e** aliphatic index, **f** secondary structure propensity and percentage content of **g** aromatic or **h** short residue side chains. **i** Partition coefficient analysis reveals that APRs identified by Cordax are primarily soluble sequences compared to easy to identify sequences of joint prediction. On the other hand, APRs that remain hard to detect are characterised by higher solubilities. Solubility regions (vi very insoluble, i insoluble, n neutral, s soluble, vs very soluble) are shown as coloured backgrounds. Significant differences were computed using one-way ANOVA with multiple comparison. Violin plots represent the kernel probability densities of the data with the median, upper and bottom quartiles.

To further explore the capabilities of our method, we composed a map of the known amyloid-forming sequence space using t-distributed stochastic neighbour embedding (t-SNE) for dimensionality reduction (Fig. 6a). As input, we used a 20-dimensional parameterisation vector describing all newly identified amyloidogenic peptides merged to the known amyloid-forming hexapeptide sequences in WALTZ-DB, in terms of their basic physicochemical properties and amino acid composition, as well as prediction outputs derived from Cordax and other high specificity predictors. t-SNE mapping pinpointed clear areas of sequence space where Cordax correctly identifies amyloid propensity (purple colour in Fig. 6a), which primarily extend towards regions that remain unpredicted (shown in black) and seclude from a large base of sequences identified by multiple methods, including Cordax (cyan colour). Clustering analysis (Fig. 6b) performed using physicochemical properties (Figs. 6c–e), secondary structure propensities (Fig. 6f) and side chain size distributions (Fig. 6g, h) identifies that this common base of by-now easy to predict APRs are characterised by high hydrophobicity, strong *β*-sheet propensity and a high relative content of aliphatic side chains (cluster 1 in Fig. 6b), still echoing the initial discovery of APRs by these features^6^. Cordax explores regions adjacent to this with a higher content of shorter side chains (clusters 2 and 5). Notably, amyloid nucleators of this composition are an invaluable resource for amyloid nanomaterial designs with elastin-like properties, are enriched in functional amyloids and have also been linked to ancestral amyloid scaffolds in early life^42–45^. A similar trend in amino acid composition has also been reported for proteins that form condensates through phase transition, such as TDP-43 and FUS^16,18^. Low complexity regions (LCRs) that are enriched in short side chains, such as Gly or Ala, have been shown to drive phase separation, often as an intermediate event towards fibrillation, particularly in polar LCRs with lower aliphatic content and strong disorder or α-helical propensities, such as the sequences discovered in cluster 5^17,46^. Further to this, Cordax provides significant advancement by traversing in areas with a higher content of negatively or positively charged regions (clusters 3, 4, 6 and 7, respectively). Charged residues often act as gatekeepers that directly disrupt aggregation or modulate it by flanking APRs within protein sequences^47^. Based on this premise, most sequence-based predictors negatively correlate net charge to protein aggregation and have increased failure rates when identifying such amyloid-forming stretches. On the other hand, sequences with a high content of aromatic side chains are relatively easy to identify (clusters 9a and 9b), following several lines of evidence supporting their role in amyloid fibril formation^48^. Cordax also pushes forward into less well-charted areas of amyloid sequence space, e.g. exploring clusters with high *α*-helical content (cluster 10) and overall a low content of aliphatic amino acids (clusters 5, 6, 7, 8 and 9b). These regions also reveal the scope to improve the method, as in particular, the region with high disorder propensity (cluster 11) still contains many false negatives, in spite of the ability of Cordax to partially pick up a minority of sequences. Interestingly, a closer look at the partition coefficients of the known amyloid sequence space reveals that although Cordax takes a significant step forward towards the right direction, these APRs remain very hard to identify as they are characterised by even higher solubility values (Fig. 6i). Similar charting of the amyloid sequence space is achieved by using uniform manifold approximation and projection (UMAP) for dimensionality reduction (Supplementary Fig. 3a and b), while PCA analysis highlights that CORDAX slowly infiltrates the sequence space of higher solubilities (Supplementary Fig. 3c and d). Overall, dimensionality reduction transformation highlights that structural compatibility can overcome typical sequence propensities as a pivotal driver of aggregation nucleating sequences and suggests that under the proper conditions, the boundaries currently considered compatible to protein amyloid-like assembly are potentially far wider than previously expected.

### Cordax predicts the structural layout and topology of fibril cores

**Fig. 7:**
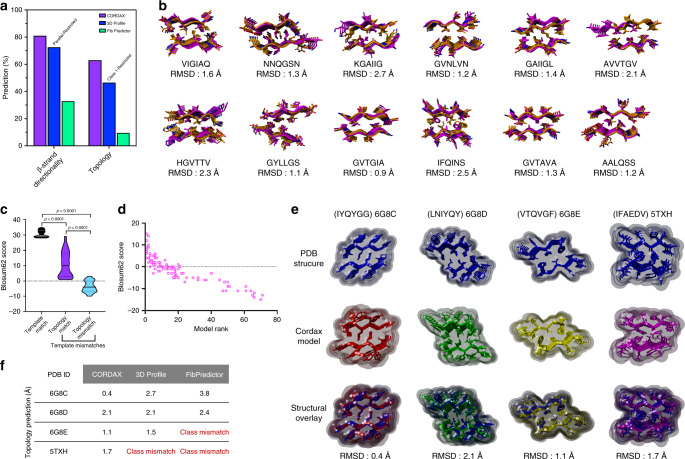
High-precision recognition of amyloid fibril structural architectures using Cordax. **a** Prediction accuracy comparison of Cordax to the only publicly available structural predictors, Fibpredictor and 3D-profile. For comparison, methods were run against a non-redundant sequence set extracted from amyloid-forming peptide interfaces (*n* = 73 templates). **b** Model topologies, predicted by applying Cordax (shown in orange), strongly superimpose to matching solved structural layouts of amyloidogenic nucleators (shown in magenta), as indicated by the reported minor RMSD values. **c** Sequence identity contribution for template selection during cross-threading analysis of the Cordax structural library. Alignment scores for selected models matching the template sequences compared to mismatching template selections of similar or different topological layouts (*n* = 73 templates). **d** Alignment scores of the APRs newly identified by Cordax to the sequence of the selected templates, plotted against their corresponding model ranks (*n* = 96 sequences threaded). **e** Structural alignment of Cordax outputs to experimentally determined 3D structures. Models were calculated for three aggregation prone sequences derived from CsgA curli forming protein (PDB IDs: 6G8C, 6G8D and 6G8E, respectively) and a peptide mutant sequence derived from A*β* amyloid peptide (PDB ID: 5TXH). Predicted topologies are overlapping representations of the experimentally determined amyloid fibril cores, **f** as displayed by a direct comparison to other software. Violin plots represent the kernel probability densities of the data with the median, upper and bottom quartiles. Source data are provided as a Source Data file.

Due to restricted availability of experimentally determined structures not included in the Cordax library, we first analysed the information derived from cross-threading analysis in order to test the performance of the tool in predicting the structural architecture of aggregation prone stretches. Among 73 unique sequences corresponding to the structural library, Cordax was able to accurately assign the correct architecture to 63%, whereas 81% was identified with proper *β*-strand orientation (parallel/antiparallel) (Fig. 7a, Supplementary Data 3 and 4). In comparison, FibPredictor^49^ correct topology allocation was limited to 9.5% of the sequences and assigned *β*-strand directionality amounted to 32.9%, while introducing an evident preference towards antiparallel architectures (Fig. 7a). Similarly, the 3D-profile method is restricted to linking all potential queries with a class 1 topology, hence was incapable of predicting alternative architectures (Fig. 7a). Structural alignment indicated that even in cases of mismatching selected templates, modelled architectures strongly superimpose to the solved structures (Fig. 7b), suggesting that Cordax identifies the correct topology with high accuracy. A closer look reveals that sequence specificity may be a modulating, yet not determining factor for this selection process. Steric perturbations can be introduced due to restrictions deriving from closely interdigitating side chains within the packed interfaces, therefore, key residue positions can be bound to the overall stability of certain structural topologies and decrease the acceptable sequence space that can accommodate energetically favourable interactions. This is highlighted by the sequence similarity observed between topological matches (Fig. 7c, Supplementary Data 4). On the other hand, topologically different model selections could also be a consequential outcome of amyloid polymorphism. The observed sequence redundancy of the Cordax library illustrates that APRs can form amyloid fibrils with distinct morphological layouts^50–52^, a notion that is also supported by the common morphological variability of aggregates formed at the level of full-length amyloid-forming proteins^53,54^. The modulating role of sequence dependency was also evident for the 96-peptide screen. A ranked analysis of the output models indicated that templates with higher alignment scores were not crucial for the topology selection process, although could often correspond to the favourable architectures (Fig. 7d), thus highlighting that the structural predictions of Cordax are relatively unbiased in terms of the sequence space composing the structural templates.

The accuracy of the tool was also cross-referenced against experimentally determined structures of fibril cores not included in the structural library. We utilised the recently solved structures of parallel fibril-forming segments derived from the major curli protein CsgA^55^, as well as an anti-parallel polymorphic APR variant segment derived from the amyloid-*β* peptide^56^. Compared to other structural predictors, only Cordax could invariantly predict the correct architecture for every steric zipper as the closest representation of the experimentally determined reference structures (Fig. 7e and f). This performance can only improve as the fragment library expands, so we aim to update it at regular intervals, providing there is a noticeable increase in solved structures in the future.

## Discussion

The number of amyloid structures in the protein databank has been steadily increasing over the last two decades. It has now achieved a number (>80) that was reached for globular proteins at the beginning of the 1980s and that then triggered the first developments of template-based modelling methods including homology-based and threading (or fold recognition) in an attempt to estimate the versatility of individual folds and discover novel folds in a more directed manner. Similarly, we here developed Cordax, an exhaustively trained regression model that leverages a substantial library of curated amyloid template structures combined with machine learning. Cordax uses a logistic regression approach to translate structural compatibility and interaction energies into sequence aggregation propensity and is therefore unconstrained by defined sequence tendencies, such as hydrophobicity or secondary structure preference that direct most sequence-based predictors. As a result, we discovered unconventional amyloid-like sequences, including sequences with low aliphatic content, high net charge or sequences with low intrinsic structural propensities. Clustering amyloid sequences by t-SNE two-dimensional reduction revealed the substructure of amyloid sequence space. Apart from a large cluster corresponding to sequences found in the hydrophobic core of globular proteins, we also found clusters corresponding to surface-exposed amyloid sequences in globular proteins, small aliphatic functional amyloids, N/Q/Y prions, strongly helical and intrinsically disordered sequences which could be compatible with liquid–liquid phase responsive sequences. Our analysis highlights the discovery of highly soluble, yet amyloid-forming, sequences and suggests that the largest portion of the remaining uncharted amyloid sequence space is hidden in this corner (Fig. 6a and i). Indeed, most archetypal hydrophobic APR sequences have low intrinsic solubility. As a result, low solubility and aggregation propensity are properties that are often wrongly used interchangeably. It is important to differentiate between the initial solubility and aggregation propensity of a peptide, as soluble monomeric sequences can often self-assemble, at later time points, into insoluble amyloid fibrils. The APRs that are newly discovered by Cordax are often highly soluble in their monomeric form, even more than the already known polar APRs from the yeast prions, as they contain many charged and polar residues, yet surprisingly can still assemble into amyloids. Overall, our approach demonstrates that the increasing structural information on amyloids now allows for more fine-graded structural rule learning of the amyloid state.

Recent developments in microcrystal electron diffraction have enabled structural determination from nanocrystals that are not typically suited for traditional X-ray diffraction and have provided significant insights on the polymorphic architectures of amyloid fibrils^57^. In this line, the emergence of cryo-EM has been pivotal in determining features of amyloid fibril polymorphs^58^, complementing earlier efforts developed using solid-state NMR spectroscopy^53,59^. Notably, these structures represent snapshots of the kinetic cores of aggregation or end-state morphologies of amyloid fibrils and therefore provide limited information on the underlying aggregation pathways and toxicity-related effects of amyloids. On the other hand, the growing number of high-resolution cryo-EM structures has highlighted the in vivo structural diversity of amyloid fibrils^60^, whereas steric zippers have been recently used for the development of targeted therapeutics^61–63^. However, determining the structural layout of amyloid fibrils still remains challenging. Cordax attempts to provide a cost-effective complementary powerful computational alternative that can be operated without any required scientific expertise necessary to apply the intricate technical approaches. Apart from its function as an aggregation predictor, the tool is uniquely poised to provide detailed complementary structural information on the putative amyloid fibril architecture of identified APRs. Users can utilise the method to structurally characterise identified APRs by classifying their overall specific topological preferences, including *β*-strand directionality and key residue positions that are integral parts of the amyloid core. The latter information is imperative for efforts focused on understanding the underlying mechanisms that dictate amyloid-related diseases or the formation of functional amyloids, but can also have an immense impact on the design of applied nano-biomaterials^64^, targeted amyloid inducers^65^ or counteragents, following the increased interest in the development of structure-based inhibitors of aggregation^61–63^.

## Methods

### Regression model training

In previous work we synthesised and explored the aggregation potential of 940 peptide sequences derived from both functional and pathological amyloid-forming proteins, which were supplemented with additional data on 462 hexapeptides derived from other published sources to develop WALTZ-DB 2.0^32^, the largest public comprehensive repository of experimentally defined amyloidogenic peptides. In total, 1402 hexapeptide sequences from WALTZ-DB were modelled on the 140 backbone structures of the Cordax library, leading to the generation of 196,280 models. The thermodynamic stability of each model (Δ*G*, kcal mol^−1^) was calculated using FoldX and fed into a logistic regression model (Fig. 2c). This model was used to distil the aggregation propensity from the free energy values. Towards this end, from the calculated Δ*G*s, we isolated 50 representative energies using a recursive feature elimination algorithm (using the RFE module of the SciKit-learn python package^33^ and selecting for the set of templates that maximised the AUC). As a result, each sequence is described with a 50-dimensional vector. Next, the data were transformed in order to be constrained in a scoring range between 0 and 1, using a Min/Max scaling algorithm. The regression model was trained with L2 penality and regularisation strength (*C*) equal to 1. Both scaling of the estimated Δ*G* and the machine-learning model were developed using the SciKit-learn python package^66^.

### Model pipeline

Cordax receives a protein sequence in FASTA format as input, which is fragmented into hexapeptides using a sliding window process. Sequences are then threaded against the fragment library utilising FoldX and the derived free energies are translated into scoring values for every peptide window. An energetically fitted model is selected as the closest representative of the overall topology of the amyloid fibril core for each predicted window and is provided as output in standard PDB format to the users (Fig. 2c). An amyloidogenic profile is generated by scoring every single residue of the input sequence with the maximum calculated score of the corresponding windows, followed by a binary prediction for every segment. Finally, calculated energies are stored automatically in a growing local database and can be retrieved, thus creating a ‘lazy’ interface that bypasses unnecessary computation for recurring sequence segments or future runs.

### Datasets

Performance assessment of Cordax was carried out utilising two individual data sets for peptide and protein aggregation propensity detection. Further validation of the method was performed against an independent subset screen of 96 hexapeptides sequences.

For peptide aggregation propensity, we used a dataset of 1402 non-redundant hexapeptides contained in the WALTZ-DB 2.0 repository^32^. This database is the largest currently available resource of experimentally characterised amyloidogenic peptides. It contains annotated peptide entries that are distributed in shorter subsets and extracted from literature^22,23,67–69^, in addition to peptides with experimentally determined amyloid-forming properties. As a result, it has been widely used as a validation set for several aggregation predicting tools^21,23,67,70,71^.

Collected in 2013, reg33 is a standard dataset for estimating the performance of aggregation propensity prediction in protein sequences^25^. It contains regional annotation of aggregating segments identified for 34 well-known amyloidogenic proteins. The annotation is assigned on a residue basis, thus containing 1260 residues in defined APRs and 6472 residues located in non-aggregating segments.

Last, we compiled a set consisting of 96 hexapeptide segments derived from potentially mis-annotated non-amyloidogenic regions of the reg33 dataset that were predicted as aggregation-prone segments after applying Cordax. Peptide segments were filtered for potential overlaps to the WALTZ-DB 2.0 set (Supplementary Data 2).

### Comparative analysis

Binary classification was utilised to determine performances of calculated aggregation propensities per hexapeptide fragment or per residue. As a result, predictions can be classified by comparison to experimental validation into true positives (TP), true negatives (TN), false positives (FP) and false negatives (FN), respectively. Performance is evaluated using the following metrics:

1 $${\mathrm{{Accuracy}}} = \frac{{\mathrm{{{TP + TN}}}}}{\mathrm{{{{TP + TN + FP + FN}}}}}$$

2 $${\mathrm{{Precision}}} = \frac{{\mathrm{{{TP}}}}}{\mathrm{{{{TP + FP}}}}}$$

3 $${\mathrm{{Sensitivity}}}\,({\mathrm{{Recall}}}) = \frac{{{\mathrm{{TP}}}}}{{{\mathrm{{TP + FN}}}}}$$

4 $${\mathrm{{Specificity}}} = \frac{{{\mathrm{{TN}}}}}{{{\mathrm{{TN + FP}}}}}$$

5 $$F1 = 2\,\times\frac{{({\mathrm{{Precision}}}\,\times\,{\mathrm{{Recall}}})}}{{({\mathrm{{Precision + Recall}}})}}$$

6 $${\mathrm{{MCC}}} = \frac{{({\mathrm{{TP}}}\,\times\,{\mathrm{{TN - FP}}}\,\times\,{\mathrm{{FN}}})}}{{\sqrt {\left( {\mathrm{{{TN + FN}}}} \right)\left( {\mathrm{{{TN + FP}}}} \right)\left( {\mathrm{{{TP + FN}}}} \right)({\mathrm{{TP + FP}}})} }}$$

### Peptide synthesis

Peptides derived from the Cordax validation set were synthesised using an Intavis Multipep RSi solid phase peptide synthesis robot. Peptide purity (>90%) was evaluated using RP-HPLC purification protocols and peptides were stored as ether precipitates (−20 °C). Peptide stocks were initially treated with 1,1,1,3,3,3-hexafluoro-isopropanol (HFIP) (Merck), then dissolved in traces of dimethyl sulfoxide (DMSO) (Merck) (<5 %), filtered through 0.2 μm filters and finally in milli-Q water to reach a final concentration of 200 μM or up to 1 mM for dye-negative peptides. Dithiothreitol (DTT) (1 mM) was included in solutions of peptides spanning cysteine or methionine residues. All peptides were incubated at room temperature for a period of 5 days on a rotating wheel.

### Thioflavin-T and pFTAA-binding assays

Amyloid aggregation was monitored using fluorescent spectroscopy-binding assays. Th-T (Sigma) or pFTAA (Ebba Biotech AB) was added in half-area black 96-well microplates (Corning, USA) at a final concentration of 25 and 0.5 μM, respectively. Fluorescence intensity was measured in replicates (*n* = 6) using a PolarStar Optima and a FluoStar Omega plate reader (BMG Labtech, Germany), equipped with an excitation filter at 440 nm and emission filters at 490 and 510 nm, respectively.

### Transmission electron microscopy

Peptide solutions were incubated for 5 days at room temperature in order to form mature amyloid-like fibrils. Suspensions (5 μL) of each peptide solution were added on 400-mesh carbon-coated copper grids (Agar Scientific Ltd., England), following a glow-discharging step of 30 s to improve sample adsorption. Grids were washed with milli-Q water and negatively stained using uranyl acetate (2% w/v in milli-Q water). Grids were examined with a JEM-1400 120 kV transmission electron microscope (JEOL, Japan), operated at 80 keV.

### Congo red staining

Droplets (10 μL) of peptide solutions containing mature amyloid fibrils were cast on glass slides and permitted to dry slowly in ambient conditions in order to form thin films. The films were stained with a Congo red (Sigma) solution (0.1% w/v) prepared in milli-Q water for 20 min. De-staining was performed with gradient ethanol solutions (70–90%).

### Determination of peptide propensities

Surface exposure and secondary structure analysis was performed using the FoldX energy force field on the available crystal structures for acylphosphatase-2 (PDB ID:1APS), amphoterin (PDB ID:1CKT and 1HME), apolipoprotein-C2 (PDB ID:1I5J), α-synuclein (PDB ID:1XQ8), *β*2-microglobulin (PDB ID:1A1M), casein (PDB ID:6FS5), gelsolin (PDB ID:3FFN), Het-S (PDB ID:2WVN), kerato-epithelin (PDB ID:5NV6), lactoferrin (PDB ID:1CB6), prolactin (PDB ID:1RW5), major prion protein (PDB ID:1E1G), repA (PDB ID:1HKQ), serum amyloid alpha (PDB ID:4IP8), Sup35 (PDB ID:4CRN) and Ure2p (PDB ID:1HQO). Partition coefficients were calculated using PlogP, which specialises in peptides with blocked termini^72^. Structural alignment and visualisation were performed with the aid of YASARA^73^. Sequence similarities were calculated using the BLOSUM62 matrix currently available under the *Biostrings* R library. Correlation plots were generated using the *ggpairs*() function available under the *GGally* R library and ROC curves were calculated using *ROCR*.

### Dimensionality reduction analysis

A defined amyloid-forming sequence space was constructed by merging the experimentally determined amyloid sequences of the 96-peptide screen, identified by Cordax, to the amyloid sequence content extracted from WALTZ-DB. Prior to t-SNE analysis, scoring outputs using Cordax, PASTA^23^, TANGO^7^ and WALTZ^21^ were calculated for each peptide entry. Peptide description was complemented with a 20-dimensional vector using the available R package *Peptides*. All data points were reduced and embedded in 2D-space using the *Rtsne* package, with perplexity (*p* = 45), iteration steps (*n* = 5000) and learning rate (default) defined based on the initial guidelines proposed by van der Maaten and Hinton^74^. UMAP reduction was performed using the R *umap* package and three-dimensional PCA analysis was conducted using *pca3d* R package and visualised with scatter3D, respectively.

### Reporting summary

Further information on research design is available in the Nature Research Reporting Summary linked to this article.

## Supplementary information

Supplementary Information

Description of Additional Supplementary Information

Supplementary Data 1

Supplementary Data 2

Supplementary Data 3

Supplementary Data 4

Reporting Summary

## Data Availability

The source data underlying Figs. 3a–d, 4l, m and 6a, c, d are provided as a Source Data file. Other data are available from the corresponding authors upon reasonable request.

## Source data

Source Data

## Code availability

CORDAX is implemented in Python and is freely accessible for academic and non-profit users at https://cordax.switchlab.org/.
