# Supplementary material for: Structure-based machine-guided mapping of amyloid sequence space reveals uncharted sequence clusters with higher solubilities
Source: Nat Commun. 2020 Jul 3;11:3314. doi: 10.1038/s41467-020-17207-3 (PMC7335209; doi:10.1038/s41467-020-17207-3)
Supplement: Supplementary file 1 — Supplementary Information [file 41467_2020_17207_MOESM1_ESM.pdf]

Structure-based machine-guided mapping of amyloid sequence space reveals uncharted sequence clusters with higher solubilities

Nikolaos Louros et al.

Supplementary Information

Supplementary Table 1. Performance on regional detection of aggregation prone segments in the reg33 dataset using the annotation described in[1].

Predictor	Sensitivity (%)	Specificity (%)	MCC
CORDAX	25.87	89.49	0.17
WALTZ	56.43	65.42	0.16
AGGRESCAN	35.37	79.26	0.13
SALSA	69.63	47.44	0.13
MILAMP	62.33	62.80	0.19
3D profile	17.95	87.53	0.06
TANGO	13.67	95.57	0.14
Zyggregator	28.73	86.31	0.15
AMYLPRED2	38.30	83.73	0.20
PAFIG	51.75	71.43	0.18
FISH Amyloid	13.73	93.68	0.10
Fold Amyloid	20.71	86.97	0.08
PASTA 2.0 (High sensitivity)	40.87	84.95	0.24
MetAmyl (High Specificity)	39.05	83.14	0.19

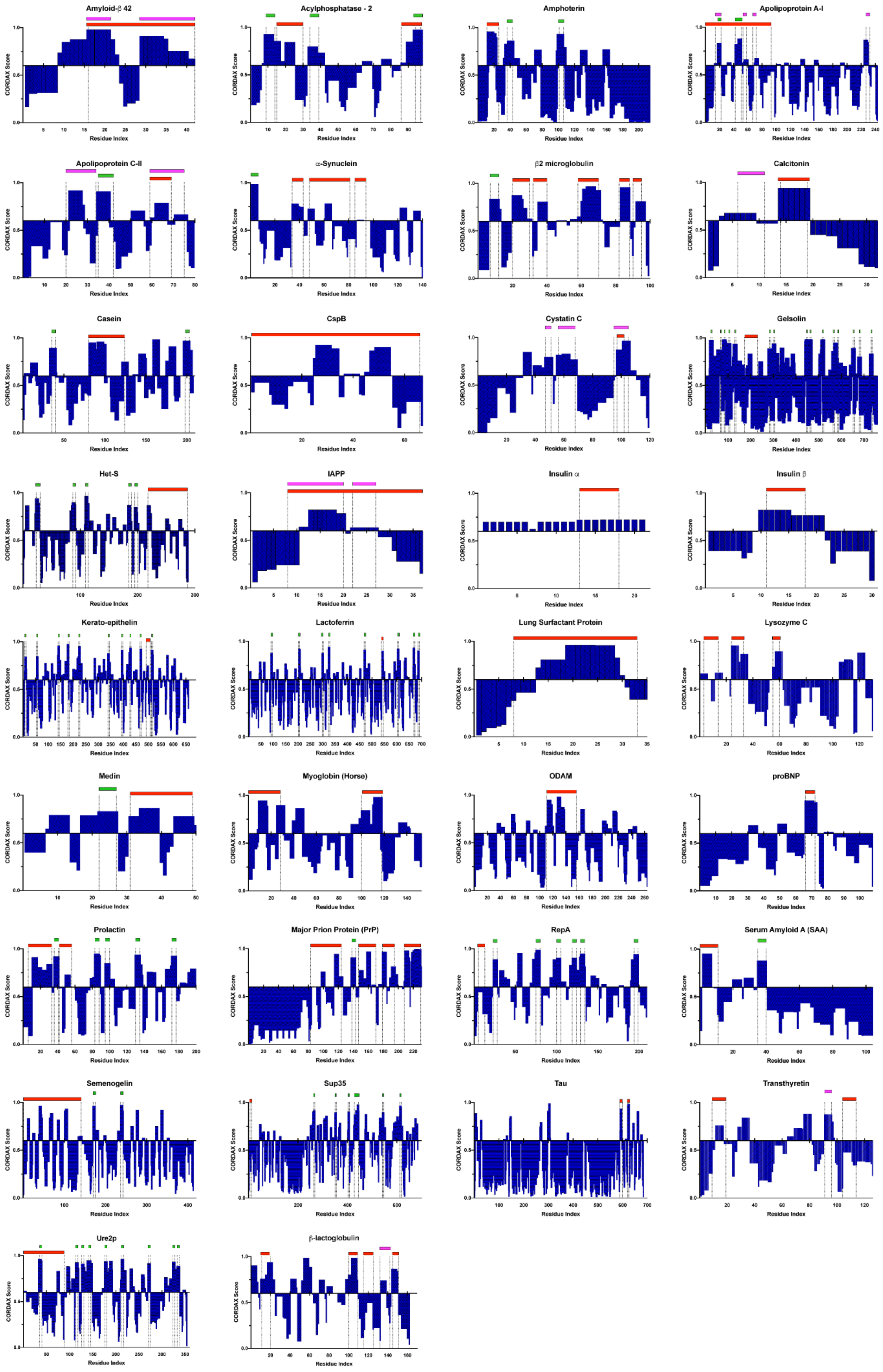
Amyloid-β 42
Acylphosphatase - 2
Amphoterin
Apolipoprotein A-I
Apolipoprotein C-II
α-Synuclein
β2 microglobulin
Calcitonin
Casein
CspB
Cystatin C
Gelsolin
Het-S
IAPP
Insulin α
Insulin β
Kerato-epithelin
Lactoferrin
Lung Surfactant Protein
Lysozyme C
Medin
Myoglobin (Horse)
ODAM
proBNP
Prolactin
Major Prion Protein (PrP)
RepA
Serum Amyloid A (SAA)
Semenogelin
Sup35
Tau
Transthyretin
Ure2p
β-lactoglobulin
CORDAX Score
Residue Index

Supplementary Figure 1. Amyloidogenic profiles of 34 amyloid-forming proteins generated using Cordax. The tool identifies most protein segments that were characterized as amyloidogenic during the initial collection[1] of the dataset (shown in red bars) and further improves once considering recent annotations of higher accuracy (shown in magenta)[2-8]. Experimentally verified aggregation prone regions strongly predicted by Cordax are highlighted by overlaid green bars.

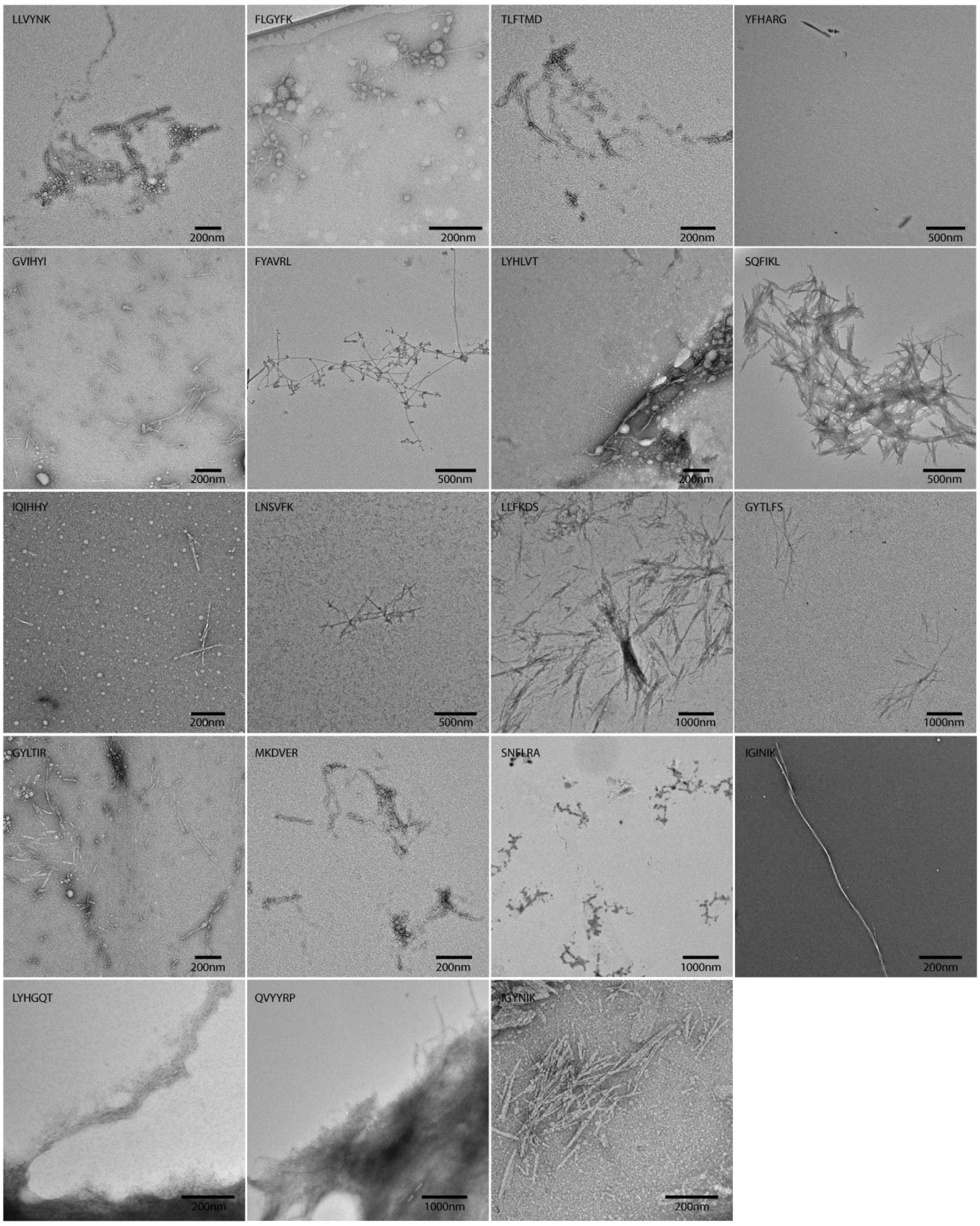

Supplementary Figure 2. Amyloid formation by peptides that fail to bind Thioflavin-T or pFTAA. Fibrils exhibit typical amyloid-like characteristics but appear shorter in length (representative images of n = 3 independent experiments).

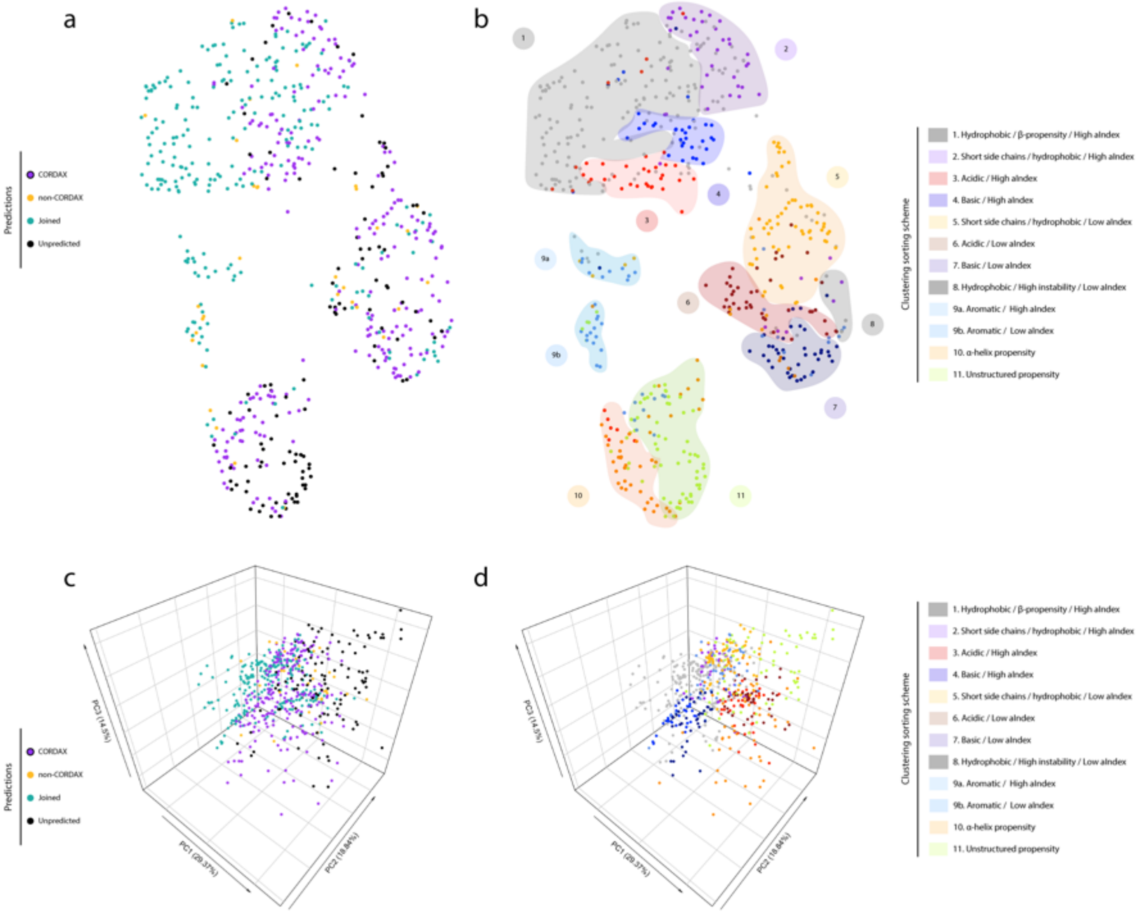

Supplementary Figure 3. UMAP and PCA analysis of the known experimentally determined amyloidogenic sequence space. (a) UMAP color-coded based on predictor performances, as in Figure 6a. (b) Clustering using the same basic physicochemical properties and amino acid composition scheme as in Figure 6b. Three-dimensional principle component analysis of the amyloid sequence space color-coded based on predictor performances (c) and (d) sequence clustering indicates that Cordax infiltrates the sequence space of higher solubilities with the exception of the high disorder propensity cluster contains many false negatives.

SUPPLEMENTARY REFERENCES

1 Tsolis, A. C., Papandreou, N. C., Iconomidou, V. A. & Hamodrakas, S. J. A consensus method for the prediction of 'aggregation-prone' peptides in globular proteins. *PloS one* **8**, e54175, doi:10.1371/journal.pone.0054175 (2013).

2 Iconomidou, V. A., Leontis, A., Hoenger, A. & Hamodrakas, S. J. Identification of a novel 'aggregation-prone'/'amyloidogenic determinant' peptide in the sequence of the highly amyloidogenic human calcitonin. *FEBS letters* **587**, 569-574, doi:10.1016/j.febslet.2013.01.031 (2013).

3 Tsiolaki, P. L., Louros, N. N., Hamodrakas, S. J. & Iconomidou, V. A. Exploring the 'aggregation-prone' core of human Cystatin C: A structural study. *Journal of structural biology* **191**, 272-280, doi:10.1016/j.jsb.2015.07.013 (2015).

4 Saelices, L. *et al.* Uncovering the Mechanism of Aggregation of Human Transthyretin. *The Journal of biological chemistry* **290**, 28932-28943, doi:10.1074/jbc.M115.659912 (2015).

5 Baxa, U. *et al.* Characterization of beta-sheet structure in Ure2p1-89 yeast prion fibrils by solid-state nuclear magnetic resonance. *Biochemistry* **46**, 13149-13162, doi:10.1021/bi700826b (2007).

6 Gross, M. *et al.* Formation of amyloid fibrils by peptides derived from the bacterial cold shock protein CspB. *Protein science : a publication of the Protein Society* **8**, 1350-1357, doi:10.1110/ps.8.6.1350 (1999).

7 Louros, N. N. *et al.* Chameleon 'aggregation-prone' segments of apoA-I: A model of amyloid fibrils formed in apoA-I amyloidosis. *International journal of biological macromolecules* **79**, 711-718, doi:10.1016/j.ijbiomac.2015.05.032 (2015).

8 Van Melckebeke, H. *et al.* Atomic-resolution three-dimensional structure of HET-s(218-289) amyloid fibrils by solid-state NMR spectroscopy. *Journal of the American Chemical Society* **132**, 13765-13775, doi:10.1021/ja104213j (2010).
