# Supplementary material for: Structure-based machine-guided mapping of amyloid sequence space reveals uncharted sequence clusters with higher solubilities
Source: Nat Commun. 2020 Jul 3;11:3314. doi: 10.1038/s41467-020-17207-3 (PMC7335209; doi:10.1038/s41467-020-17207-3)
Supplement: Supplementary file 2 — Description of Additional Supplementary Information [file 41467_2020_17207_MOESM2_ESM.pdf]

Description of Additional Supplementary Files

File Name: Supplementary Data 1
Description: List of templates incorporated in individual processing steps during generation of the CORDAX structural library.

File Name: Supplementary Data 2
Description: Amyloidogenic properties of the Cordax-predicted peptide screen.

File Name: Supplementary Data 3
Description: CORDAX cross-threading template-matching predictions. CORDAX accurately predicts both the topology and matching templates for 42.5% of the sequences derived from the structural library.

File Name: Supplementary Data 4
Description: CORDAX template-mismatch predictions. Both template and topology-defined mismatches show predominant sequence homology.
